# Supplementary material for: The impact of cell type and context-dependent regulatory variants on human immune traits
Source: Genome Biol. 2021 Apr 29;22:122. doi: 10.1186/s13059-021-02334-x (PMC8082814; doi:10.1186/s13059-021-02334-x)
Supplement: Supplementary file 2 — Additional file 2 Suppelementary Notes and Suppelementary Figures. [file 13059_2021_2334_MOESM2_ESM.pdf]

Additional file for
The impact of cell-type and context-dependent regulatory variants on human immune traits

Supplementary Notes

1 Patterns of sharing across datasets

To evaluate replication of our findings across datasets, we first verified that QTLs that were shared across DICE immune cell-types were more likely to be captured in QTL data from other datasets, particularly datasets that consist of RNA-seq from whole blood. To test this, we used Storey's π_1 statistics to estimate the proportion of eQTLs identified in DICE that are also eQTLs in the other three datasets. In particular, we partitioned the eQTLs into six groups representing the level of sharing across the major immune cell lineages sampled by DICE. We estimated that 88.2% (∼2,118 out of 2,401) of eQTLs that were shared in all six DICE immune cell groups were eQTLs in whole blood from DGN, and approximately 83% in monocyte or T cells from the BLUEPRINT consortium. By contrast, only 8.13–40.4% of eQTLs that were detected in only one DICE immune cell-type could be detected in the the DGN or BLUEPRINT data (**Fig. 1a**). In addition, when we calculated the π_1 statistics starting with only eQTLs that were specific to T-cells from DICE, we found that 17.7% were captured in T cells from the BLUEPRINT consortium. Although this rate of replication may seem low, it is 2.2-fold higher than compared to the overall proportion of cell-type-specific DICE eQTLs that were captured by BLUEPRINT T cells (**Fig. 1b**). These patterns of sharing observed are consistent with the sharing pattern and cell-type-specificity patterns of the eQTLs as inferred from the DICE dataset. We note also here that, interestingly, up to 40.4% of the cell-type-specific eQTLs identified in DICE could be replicated in the DGN dataset, suggesting that a substantial fraction of cell-type-specific eQTL effects can be detected in RNA-seq from whole blood.

2 The role of immune cells in non-immune traits

To make sense of the colocalizations found in GWAS of other non-immune traits, we reasoned that colocalized loci may either reflect a causal effect of the risk variant on disease through immune cell-types, or a causal effect of the risk variant on disease through non-immune cell-type but that is also manifested in an immune cell-type. However, if a regulatory QTL effect for the GWAS locus is only detected in an immune cell-type, then it is more likely that the GWAS variant impacts the trait through immune cell-types. Because immune cells are well known to play a role in many human traits including non-immune traits, we sought to identify trait-associated variants that likely

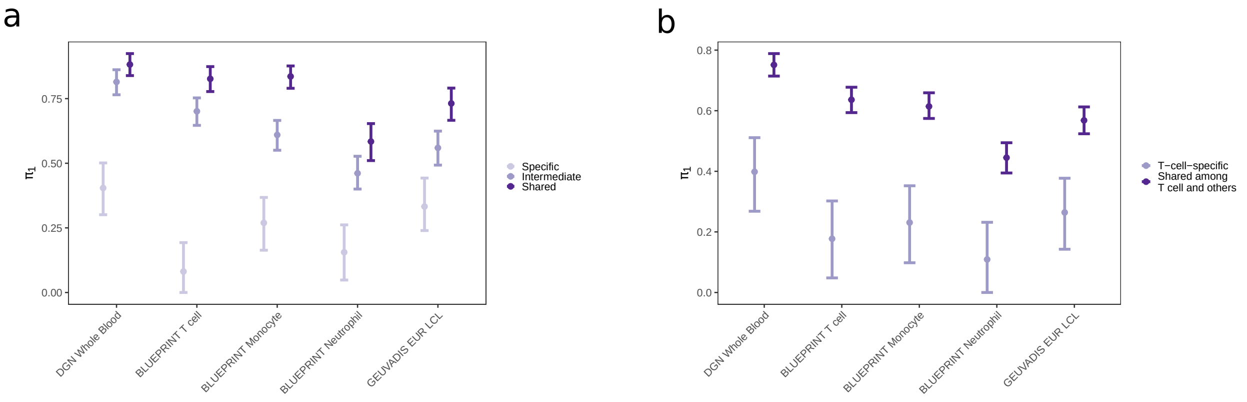

Fig. 1. Validation of eQTLs from 15 cell-types or only T cells in DICE. **(a)** Storey's π_1 statistics measuring replication of shared and cell-type-specific eQTLs in DICE across datasets. As expected, DICE eQTLs that are shared across all six cell groups are more highly replicable in other datasets than eQTLs that are specific to one cell group. Bars represent 95% confidence intervals calculated from 1,000 bootstrap samples. **(b)** Similar to **(a)**, but only eQTLs identified in T cells in DICE were validated in the other three studies.

act through immune cell-types.

Indeed, coding and regulatory variants in genes that primarily function in immune cells have been linked to autoimmune diseases, e.g. *PTPN22* in rheumatoid arthritis (RA), systemic lupus erythematosus (SLE) and type 1 diabetes (T1D) [1–3]. Interestingly, there are also many ways – perhaps less appreciated – by which immune cells impact non-immunological diseases, including coronary artery diseases (CAD) [4–6], metabolic diseases such as type 2 diabetes (T2D) [7], and neurological disorders [8, 9]. For example, microglia cells have been shown to play an important role in the development of Alzheimer's disease [10, 11]. We have also recently found that a highly significant Parkinson's disease risk locus is associated with the expression level of *LRRK2* in monocytes, but not in neuronal cells [9], suggestive of a specific effect on immune function. Thus, many, if not most, diseases are associated with a number of risk loci that function through immune cell-types.

To identify trait-associated variants that likely act through immune cell-types, we obtained eQTL p-values from relevant GTEx tissues for the DICE eQTL SNPs that colocalized with non-immune GWAS loci. Using these GTEx eQTL p-values, asked about the proportion of immune regulatory eQTLs that colocalize with non-immune GWAS loci that do not show any effects on gene expression levels in GTEx tissues that are most relevant to each trait. We chose five GTEx heart tissues as the relevant tissues for our 11 heart-related GWAS. For breast cancer, we ascertained the effect of colocalized eQTL SNPs in breast and adipose tissues. For Parkinson's disease (PD), we used the 13 GTEx brain tissues. Overall, we found that 65 of 267 (24.3%) loci that colocalized in the 14 selected GWAS are specific in DICE and BLUEPRINT immune cell data (**Supplementary File 5**, Supplementary Note 3). For example, we found that 8 of 36 eQTLs that colocalized with

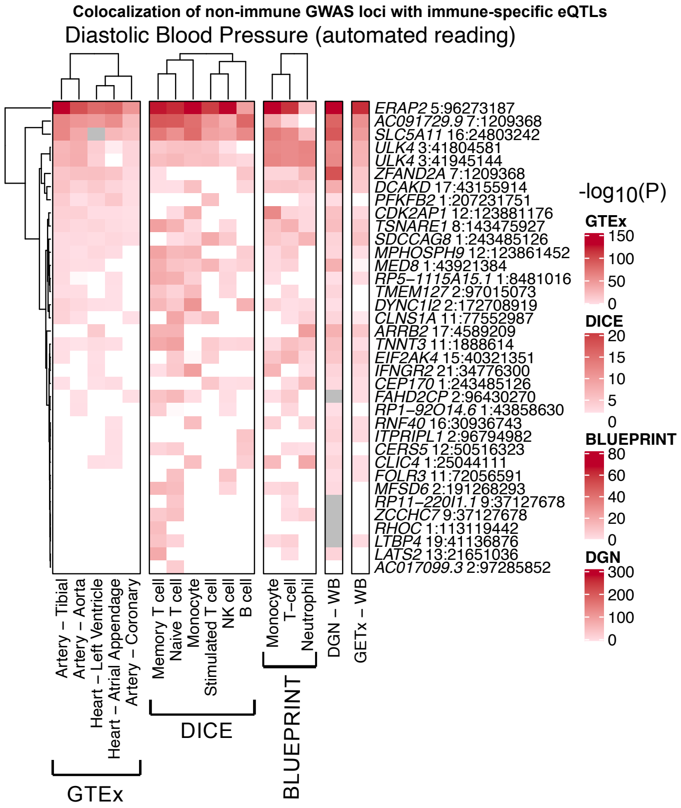

Fig. 2. Validation of eQTLs colocalized with non-immune GWAS in relevant GTEx tissues. Heatmap of eQTL association strengths in GTEx tissues and immune cell-types for DICE eQTLs that colocalize with diastolic blood pressure loci. Several GWAS loci colocalize with eQTLs active in immune cell-types but not in heart-related tissues.

diastolic blood pressure in DICE are not significant in any of the five heart related tissues (P-value > 0.05 / 5) (**Fig. 2**). Of note, two genes with colocalized eQTLs, *FOLR3* and *LTBP4*, were significantly associated with gene expression levels in GTEx whole blood, suggesting that they indeed likely function through the immune cells. Interestingly, this possibility is further supported by studies that have shown that *FOLR3* is down-regulated in peripheral blood of patients with hypertension [12, 13].

3 Colocalization of immune regulatory QTLs with non-immune trait GWAS loci

We found that no more than 30% GWAS loci for T2D, eGFR and height colocalized with our QTLs in the BLUEPRINT dataset, whereas more than 50% AD GWAS loci colocalized with a BLUEPRINT QTL. This is consistent with the known role of immune system in AD etiology. The number of genetic loci below a given p-value cutoff varies between our GWASs due to reasons

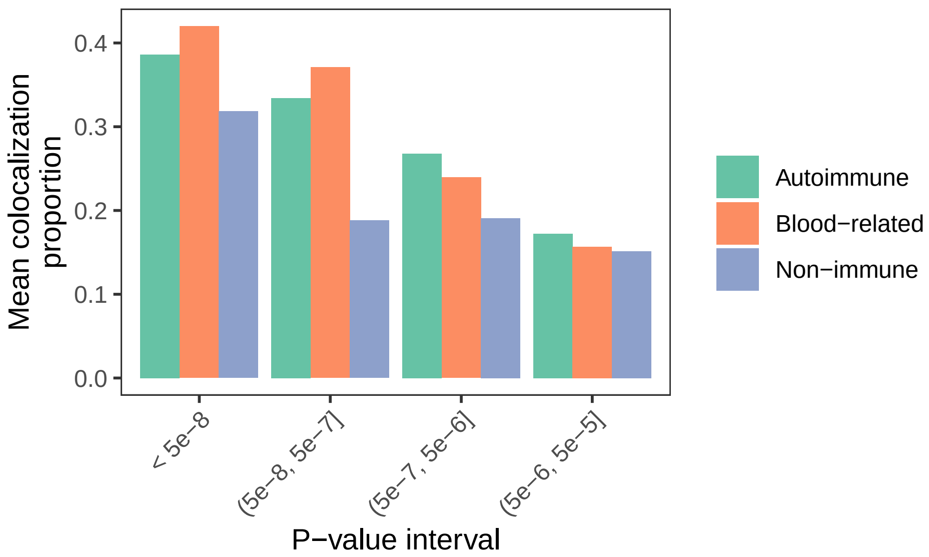

Fig. 3. Mean colocalization rates for 72 GWAS stratified by P-value bins.

including power and the genetic architecture of diseases. We observed that GWAS loci with lower p-values were more likely to colocalize with QTLs. To rule out the possibility that the difference in colocalization percentages between our autoimmune and non-autoimmune GWASs is due to differences in sample sizes (and therefore p-value distribution), we also calculated the proportion of colocalized loci binned by p-values of GWAS lead SNP. In this analysis we included GWAS lead SNPs with p-values below 10^{-5}. We found that at all p-value bins, the median of percentage of colocalized loci is higher for autoimmune diseases than non-autoimmune traits, and this difference is larger at higher p-value bins (**Fig. 3**). Interestingly, while many colocalized genes in different autoimmune diseases were shared, indicating partially overlapping disease etiology, they rarely overlapped with colocalized genes in non-autoimmune traits.

4 Underestimation of colocalization using COLOC

We found that the number of cell-types in which a GWAS locus colocalizes with an eQTL is generally smaller than the number of cell-types in which that same eQTL is inferred to be active. We speculated that this discrepancy results from the variation in the posterior probabilities of colocalization computed by `COLOC`, owing to inherent noise in estimating the effect sizes and statistical significance of eQTLs. In support of this, we found a gene *RNASET2*, whose eQTLs colocalized with a CD risk locus in 7 out of the 13 cell types analyzed (PP4 ranges between 0.79 and 0.99), but whose eQTLs were inferred to be active across all 13 cell-types (**Fig. 4**). We found that in the colocalized cell-types, the lead eQTL SNP was also the lead GWAS SNP. In the 7 other cell-types, the lead eQTL SNPs did not correspond exactly to the lead GWAS SNP, but were in strong LD

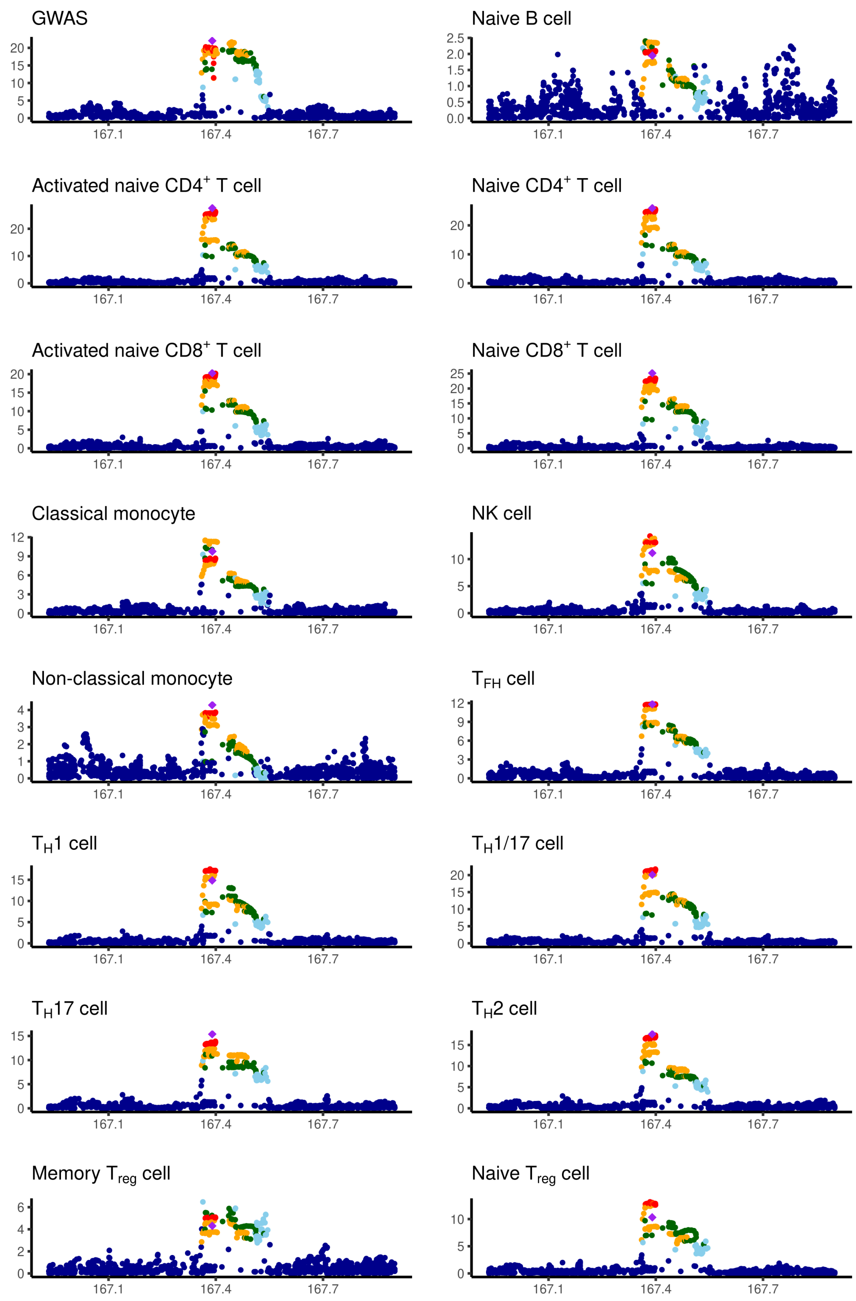

Fig. 4. LocusZoom plot for a *RNASET2* eQTL and a CD GWAS locus highlights a shared eQTL locus in DICE. The eQTL is shared among all twelve cell-types in *mash* but only colocalized in six using COLOC.

($r^2 > 0.6$). As a result of this variation, the posterior probabilities of colocalization (PP4 values) in these 6 cell-types ranged from 0.58 to 0.69, which did not pass our cutoff of 0.75. Taken together, these observations suggest that *RNASET2* eQTLs colocalize with the Crohn's disease GWAS locus in all 13 immune cell-types.

5 Robustness of colocalization

A recent study on `COLOC` [14] reported that misspecification of prior parameters can heavily impact the inferred posterior probability of colocalization. To verify the robustness of our colocalization estimates, we performed the same analyses as above using `HyPrColoc` (Hypothesis Prioritisation in multi-trait Colocalization) [15] instead of `COLOC`. Unlike `COLOC`, `HyPrColoc` calculates both SNP-level alignment posterior probabilities and a regional posterior probability. `HyPrColoc` then uses the product of the SNP-level alignment posterior probabilities and the regional posterior probability as the colocalization posterior probability. It further applies non-uniform priors to SNPs in a given genomic locus, which was proposed to be more conservative than `COLOC` [15]. We found that the posterior probabilities calculated in `HyPrColoc` were highly correlated (Spearman's $\rho = 0.86$) to the posterior of colocalization (PP4) estimated using `COLOC`, but were consistently lower (**Fig. 5a**). Using the posterior probability cutoff recommended by the authors (0.25), we were able to replicate all colocalized signals identified using `COLOC`. Indeed, `HyPrColoc` found the same number or slightly more colocalized loci when compared to `COLOC` (mean: 44% compared to 40%; **Fig. 5b**). Interestingly, we found that `COLOC` and `HyPrColoc` yield identical results when the `COLOC` PP4 cutoff was lowered to 0.5 (**Fig. 5a**). Our re-analysis of colocalization using `HyPrColoc` therefore suggests that our initial `COLOC` results are robust to assumptions on the prior distribution of colocalization probabilities. We thus performed all downstream analyses based on `COLOC` colocalization status.

To better understand the effect of a fixed PP4 cutoff on over-estimating cell-type-specificity of colocalization, we categorized all gene-cell pairs tested for colocalization by (i) whether the eGene colocalizes in at least one cell-type and (ii) whether the eGene is shared in at least four cell categories in DICE. We then compared the PP4 values of these genes in each cell-type. We found that when the eGenes are shared across cell-types, the COLOC PP4 values are larger than when the eGenes are not shared for uncolocalized gene-cell pairs. By contrast, this difference was much smaller for colocalized gene-cell pairs (**Fig. 6**). While these observations suggests that our PP4 cutoff should perhaps be lowered, we found that lowering the PP4 cutoff does not solve the overestimation issue as PP4 will be inevitably smaller than any reasonable cutoff in some cell-type. We interpret these findings to support the possibility that the cell-type-specificity of eQTL-GWAS loci colocalization is often overestimated.

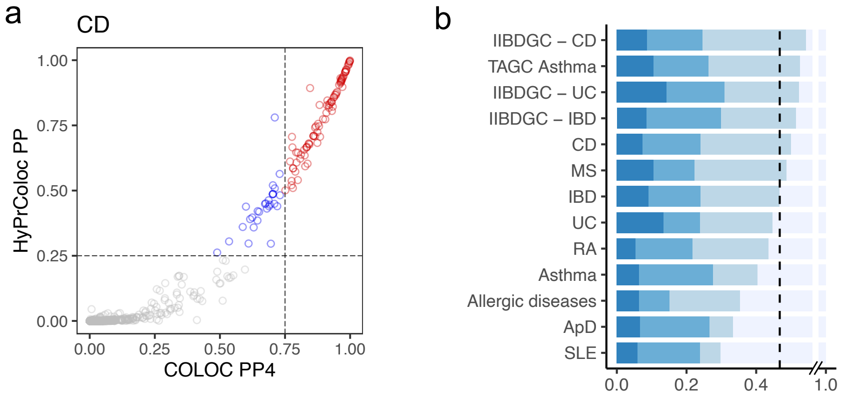

Fig. 5. Comparison of `HyPrColoc` and `COLOC` for CD GWAS. (a) Colocalization posterior probabilities from `HyPrColoc` and `COLOC` are highly correlated. Red: colocalized in both `COLOC` and `HyPrColoc`; blue: only colocalized in `HyPrColoc`; grey: not colocalized. Dashed lines: posterior probabilities used in each method. Similar patterns for other autoimmune were also observed. **b** The rate of colocalization is on average 44.9% for the 13 autoimmune diseases analyzed using `HyPrColoc`. AE GWAS was not included in `HyPrColoc` analysis because SNP effect sizes were not available in the summary statics.

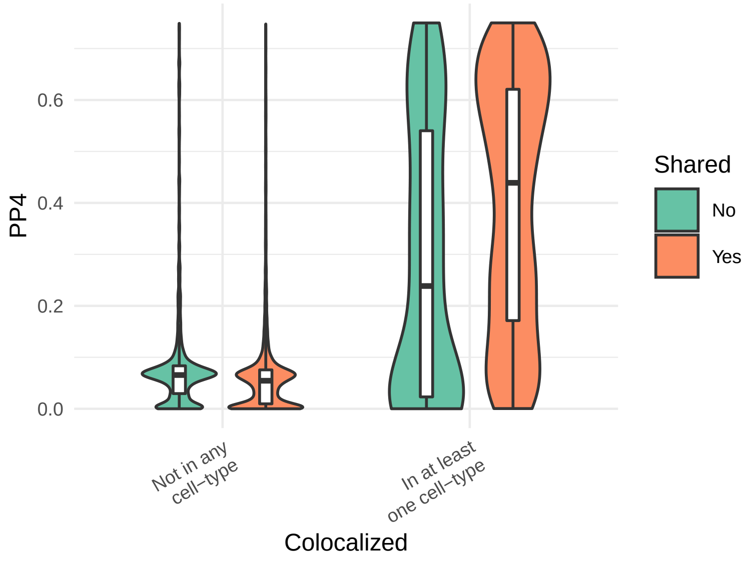

Fig. 6. Shared eGenes that colocalized in at least one cell-type tend to have larger PP4 in cell-types that do not colocalize. Each entry in the plot represents the PP4 value of an eGene with a GWAS locus in a given cell-type in DICE. Not in any cell-type: the eGenes do not colocalized in any cell-type; In at least one cell-type: the eGenes colocalized in at least one cell-type, but may be uncolocalized in other cell-types (PP4 < 0.75). Only uncolocalized tests were included in the plot. Shared: if an eGene is significant in 12 or more cell-types. Data from all 14 autoimmune GWAS were plotted together.

Supplementary Figures

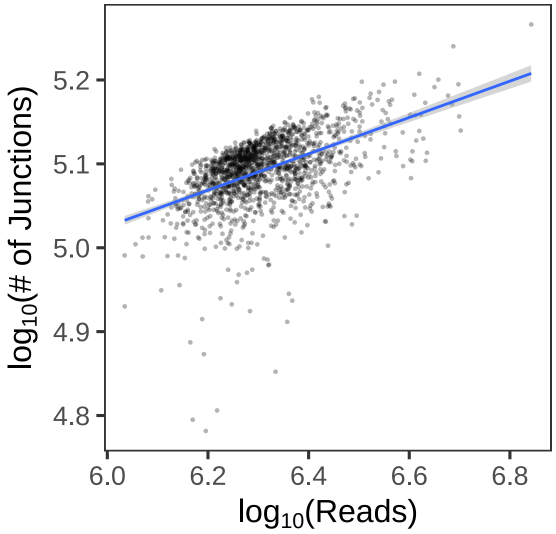

Fig. S1. Number of junctions identified in each sample is positively correlated with library sizes. Blue line represents fitted line using a simple linear model.

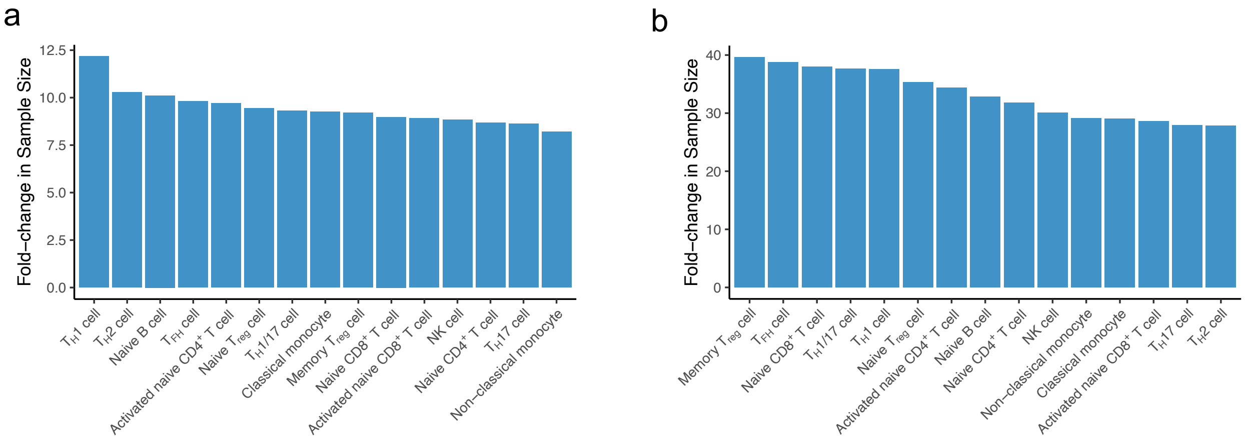

Fig. S2. Fold-change of effective sample sizes as estimated by *mash*. **(a)** Fold-change in effective sample sizes for DICE eQTLs. **(b)** Fold-change in effective sample sizes for DICE sQTLs.

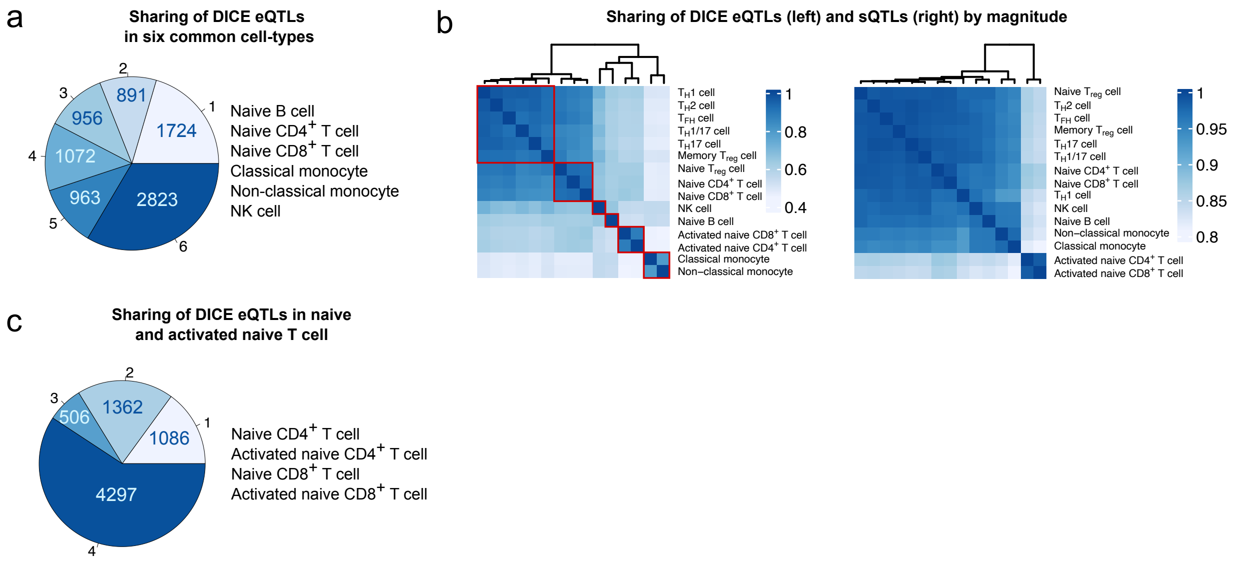

Fig. S3. Sharing of eQTLs and sQTLs using *mash* excluding genes in the HLA locus. **(a)** Fold-change in effective sample sizes for DICE eQTLs. **(b)** Fold-change in effective sample sizes for DICE sQTLs.

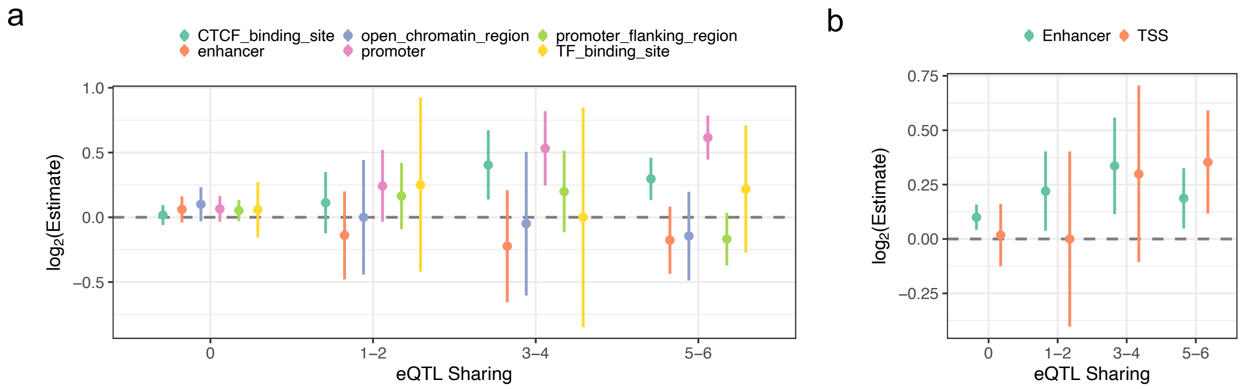

Fig. S4. Enrichment of eQTLs in regulatory elements. **(a)** Enrichment of DICE eQTLs in UCSC Regulatory Build. **(b)** Enrichment of DICE eQTLs in TSS and enhancers from Calderon et al. [16] Bars represent 95% confidence intervals for $\log_2$(Odds ratio).

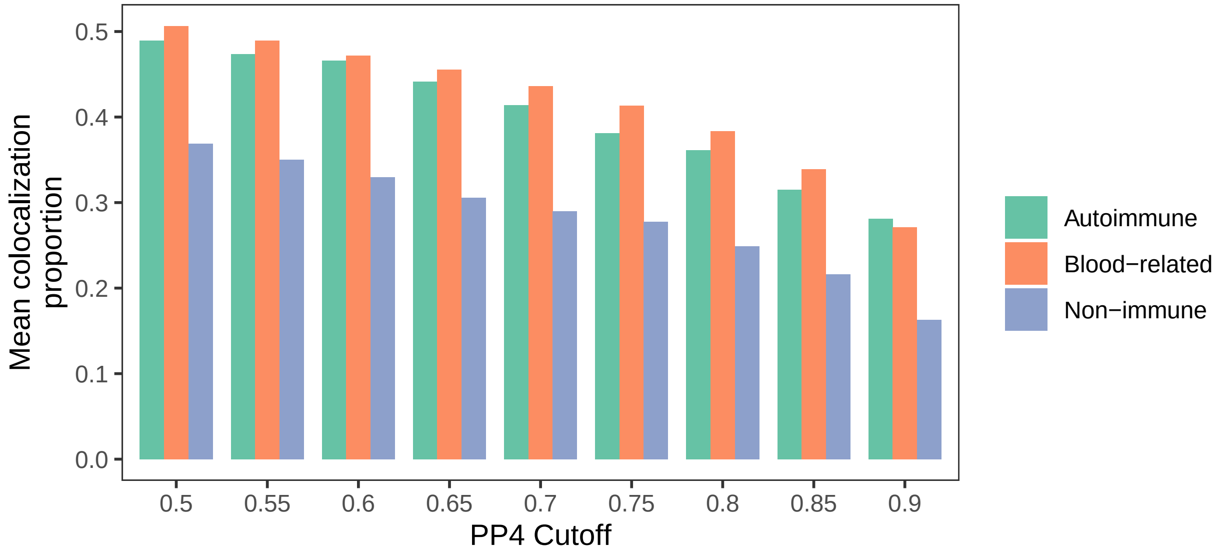

Fig. S5. Mean colocalization rates as a function of PP4 cutoff in `COLOC`.

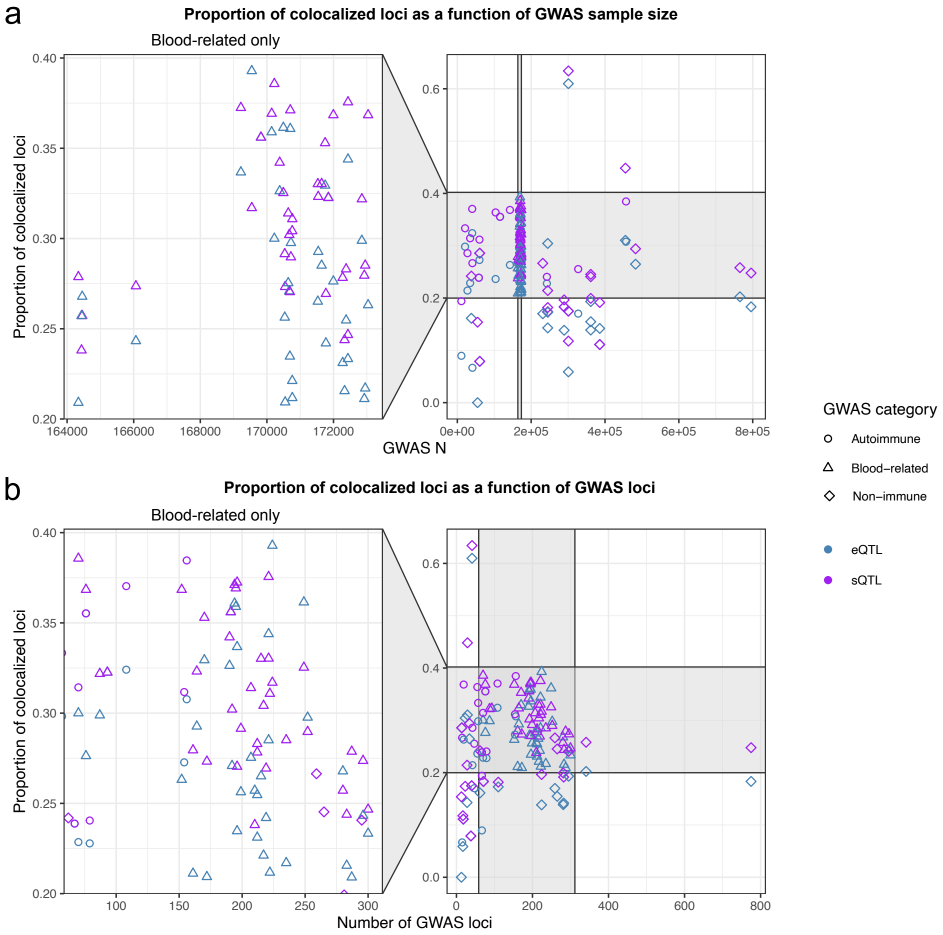

Fig. S6. Mean colocalization rates as a function of GWAS sample size and number of GWAS loci. Proportion of colocalized GWAS loci is not related to **(a)** GWAS sample size or **(b)** the number of GWAS loci.

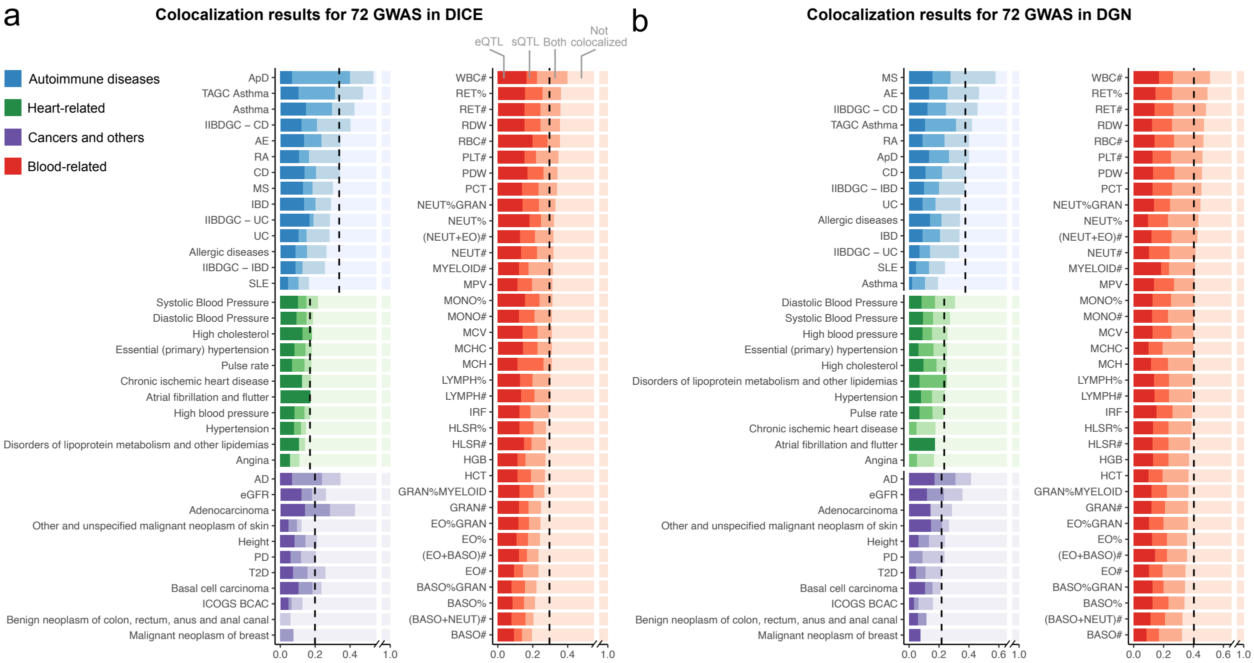

Fig. S7. Colocalization rates for 72 GWAS in DICE and DGN consortium.

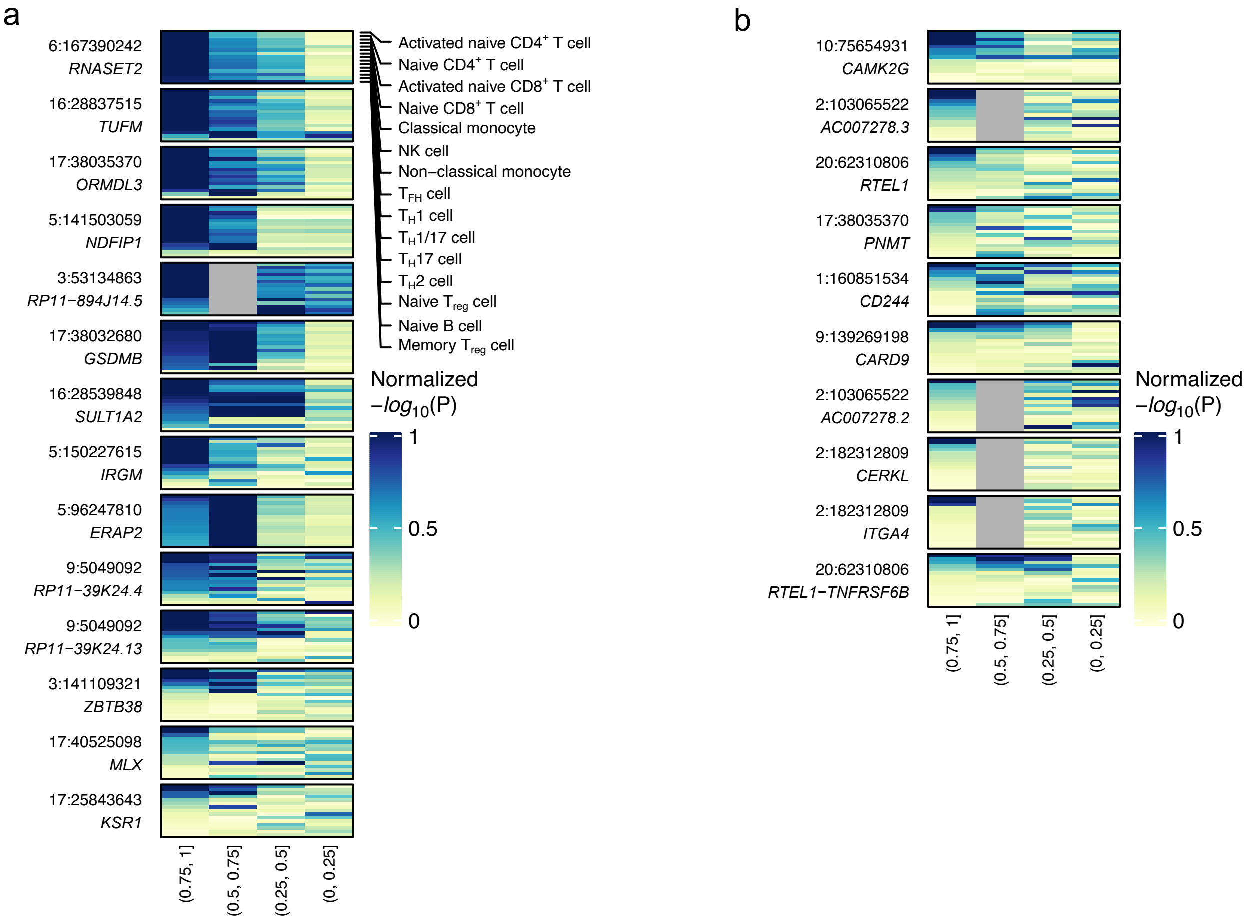

Fig. S8. **Many eGenes colocalized in CD GWAS are shared among the immune-cells.** **(a)** eQTL p-values in different LD bins at GWAS loci with colocalized eQTLs across all 15 cell-types. **(b)** Cell-type-specific colocalized eGenes show low p-values across LD bins only in a small number of cell-types.

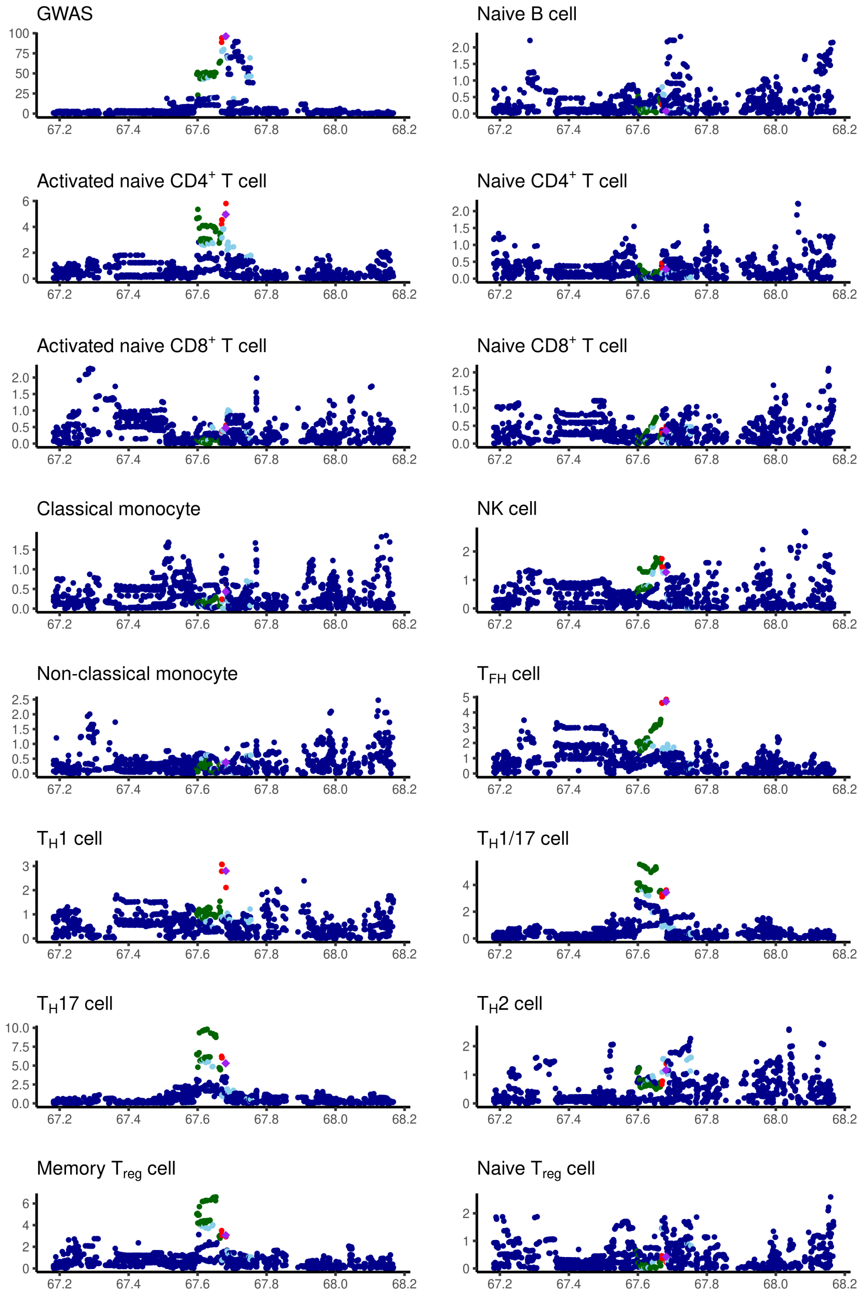

Fig. S9. LocusZoom plot for *IL23R* eQTL and a CD GWAS locus. *IL23R* eQTL colocalized with the CD GWAS locus only in activated naïve CD4$^+$ T cells.

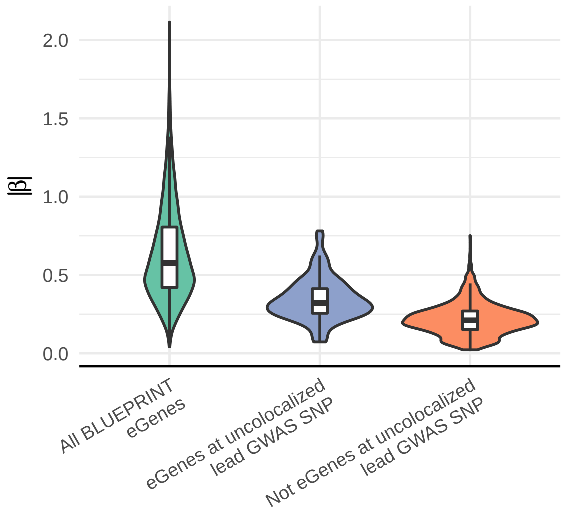

Fig. S10. Ascertainment of eQTL effect sizes at uncolocalized lead GWAS SNP. All BLUEPRINT eGenes: all eGenes from eQTL mapping that pass genome-wide multiple testing adjustment; eGenes at uncolocalized lead GWAS SNP: SNP-gene associations at lead SNPs of uncolocalized GWAS loci that were significant QTLs after multiple testing adjustment; Not eGenes at uncolocalized lead GWAS SNP: SNP-gene associations at lead SNPs of uncolocalized GWAS loci that did not pass multiple testing adjustment.

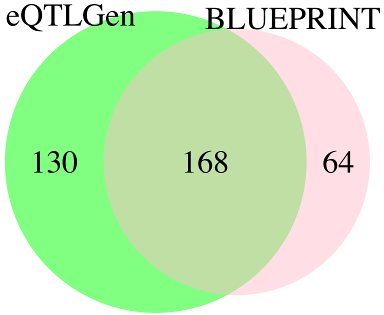

Fig. S11. Comparison of colocalized loci between eQTLGen and BLURPRINT for 14 autoimmune GWAS.

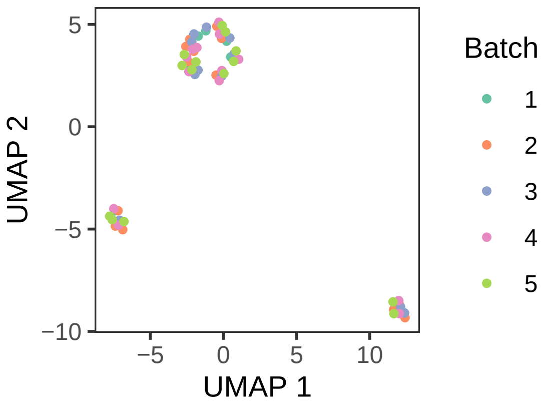

Fig. S12. Two dimensional UMAP visualization of CUT&Tag read counts in the 30k most highly variable peaks across samples. Coloring samples by batches show no clustering according to batch.

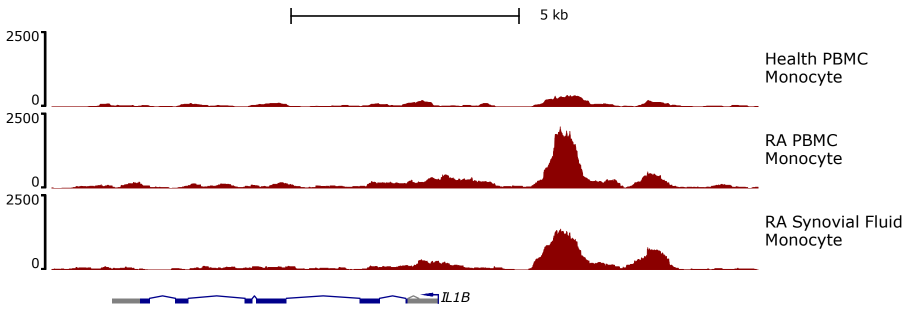

Fig. S13. Genome tracks of H3K27Ac in monocytes near *IL1B* promoter.The H3K27Ac profiles in monocytes from PBMC of RA patients near the *IL1B* promoter were more similar to that of monocytes from synovial fluids of patients than to healthy PBMC monocytes.

References

[1] Ann B Begovich et al. "A missense single-nucleotide polymorphism in a gene encoding a protein tyrosine phosphatase (PTPN22) is associated with rheumatoid arthritis". In: *The American Journal of Human Genetics* 75.2 (2004), pp. 330–337.

[2] Chieko Kyogoku et al. "Genetic association of the R620W polymorphism of protein tyrosine phosphatase PTPN22 with human SLE". In: *The American Journal of Human Genetics* 75.3 (2004), pp. 504–507.

[3] Nunzio Bottini et al. "A functional variant of lymphoid tyrosine phosphatase is associated with type I diabetes". In: *Nature genetics* 36.4 (2004), pp. 337–338.

[4] Paul M Ridker et al. "Comparison of C-reactive protein and low-density lipoprotein cholesterol levels in the prediction of first cardiovascular events". In: *New England journal of medicine* 347.20 (2002), pp. 1557–1565.

[5] John Danesh et al. "C-reactive protein and other circulating markers of inflammation in the prediction of coronary heart disease". In: *New England Journal of Medicine* 350.14 (2004), pp. 1387–1397.

[6] Nasimudeen R Jabir et al. "Reduction in CD16/CD56 and CD16/CD3/CD56 natural killer cells in coronary artery disease". In: *Immunological investigations* 46.5 (2017), pp. 526–535.

[7] Olivia Osborn and Jerrold M Olefsky. "The cellular and signaling networks linking the immune system and metabolism in disease". In: *Nature medicine* 18.3 (2012), p. 363.

[8] Yang Shi and David M Holtzman. "Interplay between innate immunity and Alzheimer disease: APOE and TREM2 in the spotlight". In: *Nature Reviews Immunology* 18.12 (2018), pp. 759–772.

[9] Yang I Li et al. "Prioritizing Parkinson's disease genes using population-scale transcriptomic data". In: *Nature communications* 10.1 (2019), pp. 1–10.

[10] Hadas Keren-Shaul et al. "A unique microglia type associated with restricting development of Alzheimer's disease". In: *Cell* 169.7 (2017), pp. 1276–1290.

[11] Susanne Krasemann et al. “The TREM2-APOE pathway drives the transcriptional phenotype of dysfunctional microglia in neurodegenerative diseases”. In: *Immunity* 47.3 (2017), pp. 566–581.

[12] Melvin T Korkor et al. “Microarray analysis of differential gene expression profile in peripheral blood cells of patients with human essential hypertension”. In: *International journal of medical sciences* 8.2 (2011), p. 168.

[13] Zilun Wei et al. “The transcriptome of circulating cells indicates potential biomarkers and therapeutic targets in the course of hypertension-related myocardial infarction”. In: *Genes & Diseases* (2020).

[14] Chris Wallace. “Eliciting priors and relaxing the single causal variant assumption in colocalisation analyses”. In: *PLoS Genetics* 16.4 (2020), e1008720.

[15] Christopher N Foley et al. “A fast and efficient colocalization algorithm for identifying shared genetic risk factors across multiple traits”. In: *BioRxiv* (2019), p. 592238.

[16] Diego Calderon et al. “Landscape of stimulation-responsive chromatin across diverse human immune cells”. In: *Nature Genetics* (2019), pp. 1–12.
