# Supplementary material for: The impact of cell type and context-dependent regulatory variants on human immune traits
Source: Genome Biol. 2021 Apr 29;22:122. doi: 10.1186/s13059-021-02334-x (PMC8082814; doi:10.1186/s13059-021-02334-x)
Supplement: Supplementary file 5 — Additional file 5 Review history [file 13059_2021_2334_MOESM5_ESM.docx]

Review History

**First round of review**

**Reviewer 1**

**Are you able to assess all statistics in the manuscript, including the appropriateness of statistical tests used?** Yes, and I have assessed the statistics in my report.

**Comments to author:**

Mu et al conduct an impressive suite of analyses integrating eQTL data sets with GWAS, and show the importance of splicing QTLs. The analyses are complex, but logically and well-explained. There are a lot of interesting results in this paper. Up until the RA sections, I find the results novel but also consistent with other reports. However, almost lost is an important conclusion that to understand the role of GWAS risk loci we need to study gene expression in the context of disease. This is important I think, because an argument against this is that gene expression in disease could reflect consequence (including response to treatment) rather than cause, hence the move to integrate GWAS result with eQTL results in healthy people. Is there a way to give more emphasis to this result in the title/abstract?

I have a couple of major points:

1) Since this is a paper about immune cells, I wondered what had been done about the HLA region. The only mention is in the Methods, which states "The HLA region (Chr6: 25Mb-35Mb) was excluded from colocalization." This implies that the HLA was included in the analyses up to this step. It would good to see a version of Figure 2 in the supplement excluding HLA (or Figure 2 in supplement and with HLA in main, depending on which is more interpretable - and may fit better with the coloc results). The discussion needs some discussion of HLA region, even if to acknowledge too complex to focus on.

2) An important part of the paper is a commentary on how power impacted on conclusions about eQTL sharing eg Page 7: "However, because the sample sizes of most datasets are small, we speculated that estimates of QTL effect sizes are noisy, which would generally cause studies to underestimate the levels of QTL sharing." And the section on page 12.

This reminded me of the Qi et al paper Nat Coms 2018 "Identifying gene targets for brain-related traits using transcriptomic and methylomic data from blood" DOI: 10.1038/s41467-018-04558-1. There are parallels between the studies and the conclusions even though that study focussed on eQTL across brain and increased power from blood, and here focus is immune cell types including blood.

i) That study approached the power issue through simulation and provided a statistic (MeCS) software to estimate correlation of effect size accounting for s.e. Maybe mash does something similar

ii) Their Figure 3, x axis difference in eQTL effect between two tissues vs y-axis difference in mean gene expression for the gene between two tissues was very interesting to me. It would be interesting to see if this relationship replicates. At first sight (Figure 2d) it seems not, but this is a more condensed analysis.

iii) In comparing eQTL effect sizes between two tissues, they first identified eQTL in a third tissue and used those SNPs as the basis for comparing eQTLs in the two tissues, arguing this could avoid winner's curse.

I am not suggesting a massive re-analysis as I think you have already done so much. Rather, I just wondered if this paper could offer anything to further improve interpretation. If not, then no need to consider further.

3) are all the eQTL meta-analysed results available for download (including with eQTLgen),

Minor points

1) "Interestingly, we observed that the rates of colocalization between GWAS loci and both an eQTL and an sQTL can vary substantially across traits, ranging from 4.5% for systemic lupus erythematosus (SLE) to 28.6% for basophil percentages of granulocyte (BASO%GRAN) (average: 17.2%)." This statement is hard to benchmark because of the impact of GWAS sample size. As far as I can see you emphasise consequence of eQTL sample size but not GWAS sample size, but maybe I missed.

2) Page 5:" DICE also performed in vitro activation of CD4+ and CD8+ T cells using CD3/CD28 antibodies." On first reading a reader isn't familiar with acronyms so this would be better phrased as "The DICE data set also included gene expression data after in vitro activation…"

3) Page 8: "This observation suggests that activated CD4+ and CD8+ T cells share similar gene expression programs upon activation, and that differences in ge-netic effects on gene regulation exist between activated and non-activated cells." This is interesting. Are there gene lists that could be provided. Eg the summary data behind Figure 2, with a statistic for ranking differences?

Page 14: Readers will want to be able to identify these "We observed that the top eQTLs are often in high LD with the lead GWAS SNP for multiple cell-groups (rows) when the eQTLs were determined to have shared effects. By contrast, for the eQTLs we inferred to have a cell-type-specific effect, the patterns are strikingly different as the most significant eQTLs are more likely to be in lower r2-bins in most cell-types'

Are these results available in the supplementary files.

4)Page 11. "Ten and fifteen loci colocalized with only eQTLs or sQTLs, respectively, while an additional 25 loci colocalized with both eQTLs and sQTLs. In total, 46% of loci colocalized with an eQTL, an sQTL, or both" So are the eQTL and sQTL SNPs in LD or independent?

5) Page 18. Note if DGN is part of eQTLgen

6) Fig 2b, where "Sharing of eQTLs (left) and sQTLs (right) by magnitude. " isnt very unclear what is plotted. This is where the MeCS statistic may be useful. Although I have now found in the MashR methods section.

7) "To avoid reads mapping with allelic bias, we used WASP [58] as implemented in STAR 2.6.0 by providing the corresponding genotype data. This is an important step as we found a substantial increase in the number of false positive splicing QTL due to allelic bias in read mapping." Please add another sentence to clarify further.

**Reviewer 2**

**Are you able to assess all statistics in the manuscript, including the appropriateness of statistical tests used?** Yes, and I have assessed the statistics in my report.

**Comments to author:**

Impact of cell-type and context-dependent regulatory variants on human immune traits

The authors perform systematic colocalisation analysis between several eQTL and sQTL datasets and 72 GWAS traits, with a particular focus on the autoimmune diseases. They find that many more GWAS signals colocalised with eQTLs than in previous research, and especially with sQTLs and that, moreover, that cell-specific eQTLs accounted to much fewer colocalisations than previously reported. Reasons for these discrepancies are postulated and studied. Additionally, authors investigate potential mechanisms through which the 60% of non- colocalised GWAS signals may affect disease susceptibility and mechanisms. As we are moving away from the GWAS era, the manuscript addresses interesting questions about disease causing mechanisms and systemic properties of gene expressions in multiple cell types.

Interesting results are presented. However, I have some reservations about the manuscript in its current format.

A major concern is the structure and length of the manuscript---it appears to be overly long and is hard to follow. An awful lot of work has been done, but a lack of coherent presentation obscures the main messages.

They appear to be

- greater sharing of QTLs across cell types. This is based on inference by mash, on which I have some major methodological concerns (see below).

- Stronger colocalisation of QTL and GWAS effects, particularly for autoimmune traits, driven by inclusion of sQTL data. The presentation here is very unclear. When QTLs colocalise with a % of GWAS traits, what is the denominator? The phrase "we obtained association between all colocalized eQTL SNPs" on page 11… colocalised with what? The inference that colocalisation of eQTL signals in whole blood and hypertension is used to support immune involvement in hypertension - but could it not also reflect sharing of QTLs between cell types as the authors propose?

- Limited cell state specific regulatory effects: is this not a restatement of the first major point?

Methodological concern on mash

Reading https://urldefense.proofpoint.com/v2/url?u=https-3A__stephenslab.github.io_mashr_articles_intro-5Fmash.html&d=DwIGaQ&c=vh6FgFnduejNhPPD0fl_yRaSfZy8CWbWnIf4XJhSqx8&r=Y4r1_XDXu4yYdegfBIAiGEOf3a2Qz0JY3Z5HybxTDto&m=LZRrrdfSL_l_8XAfXmLfLiDegEdNws6HJA1oOJOQwUg&s=Muc6qeq_FYZISe_BkcG7UtMo4ifmX4CtdL3_1pit928&e=

the mash instructions say

"The Crucial Rule: Step 3 (fitting the mash model) must be performed with either all the tests you performed, or - if that causes computational challenges - a large random subset of tests.

In particular, you must not use only a set of selected "significant" or "strong" tests in step 3. " Yet in the methods here, the authors say they ran mash using SNPs with the smallest p values across all tested SNPs and cell types for each feature. Does this claim of sharing persist if mash is run correctly? How does this impact all subsequent results?

Even if mash is run correctly, given that mash assumes effect estimates are correlated, does it have a tendency to over-call sharing of effects? This is important as the accuracy of mash was primarily assessed on its ability to *detect* true associations. Evidence of over-calling of sharing is apparent in Table 1 of Urbut et al. If there is over-calling, how does this impact conclusions?

Minor issues include typos and presentational issues, e.g.:

the mash method is mentioned without description in Figure 1 and is not introduced until page 6 despite its application to the problem being part of the novel contribution of the paper.

p 1 sQTL abbreviation not formally defined (should be on line 17/18) p 1 line 17/18. QTLs should be eQTLs.

p 2 line 2. Citation needed

p 3 line 26. should be "40.4% *of* GWAS loc"

Caption to Figure 1a. "four large immune RNA-seq datasets"---these were specifically previously described as datasets of different sizes with only two of them as large (p 3 lines 18-22).

Figure 1b. Would be nice to label the LHS part of the graph with DICE to make labelling self-sufficient, Grammatically incorrect main title of the graph.

p 5 line 3. eQTL abbreviation already explained on p 2 line 8, whilst sQTL has already been used without being defined.

page 6. p 6 line 18. LFSR not defined.

p 20 line 2. I refer the authors to a recent paper https://urldefense.proofpoint.com/v2/url?u=https-3A__journals.plos.org_plosgenetics_article-3Fid-3D10.1371_journal.pgen.1008720-26rev-3D2&d=DwIGaQ&c=vh6FgFnduejNhPPD0fl_yRaSfZy8CWbWnIf4XJhSqx8&r=Y4r1_XDXu4yYdegfBIAiGEOf3a2Qz0JY3Z5HybxTDto&m=LZRrrdfSL_l_8XAfXmLfLiDegEdNws6HJA1oOJOQwUg&s=dnY_shGxU5PQsqk-yoaSFx7MbuA2uk2Az-UXZ0j6lrI&e=

for analysis of sensitivity to prior parameters of the coloc method.

**Authors Response**

**Point-by-point responses to the reviewers’ comments:**

Reviewer reports:

Reviewer #1: Mu et al conduct an impressive suite of analyses integrating eQTL data sets with GWAS, and show the importance of splicing QTLs. The analyses are complex, but logically and well-explained. There are a lot of interesting results in this paper. Up until the RA sections, I find the results novel but also consistent with other reports. However, almost lost is an important conclusion that to understand the role of GWAS risk loci we need to study gene expression in the context of disease. This is important I think, because an argument against this is that gene expression in disease could reflect consequence (including response to treatment) rather than cause, hence the move to integrate GWAS result with eQTL results in healthy people. Is there a way to give more emphasis to this result in the title/abstract.

Thank you for your encouraging comments. We agree that we haven’t put a lot of emphasis on our results that highlight the promise of using disease samples to understand GWAS risk loci. This was done partly because we found our sample size (n = 6) to be rather small to make a stronger case, and partly because we are now collecting a lot more data which we think will support our hypothesis more convincingly.

That being said, we have now added two sentences in the introduction of the revised

manuscript to highlight this finding: *“Specifically, our work suggests that to understand the mechanisms underlying many GWAS risk loci, we need to study gene regulation in the context of disease. This stands in contrast to the idea that gene regulation in the disease context merely reflects the consequence of disease, or of response to treatment, which would confound the study of causal genetic mechanisms rather than help it.* ”

I have a couple of major points:

1) Since this is a paper about immune cells, I wondered what had been done about the HLA region. The only mention is in the Methods, which states "The HLA region (Chr6: 25Mb-35Mb) was excluded from colocalization." This implies that the HLA was included in the analyses up to this step. It would good to see a version of Figure 2 in the supplement excluding HLA (or Figure 2 in supplement and with HLA in main, depending on which is more interpretable - and may fit better with the coloc results). The discussion needs some discussion of HLA region, even if to acknowledge too complex to focus on.

Thank you for this comment. We added a Supplementary Figure 3 similar to Figure 2 where we **excluded** genes in the HLA locus. We did not notice a dramatic change in the sharing of eQTLs, which is expected given that only a small proportion of genes were excluded.

2) An important part of the paper is a commentary on how power impacted on conclusions about eQTL sharing eg Page 7: "However, because the sample sizes of most datasets are small, we speculated that estimates of QTL effect sizes are noisy, which would generally

cause studies to underestimate the levels of QTL sharing." And the section on page 12. This reminded me of the Qi et al paper Nat Coms 2018 "Identifying gene targets for brain-related traits using transcriptomic and methylomic data from blood" DOI: 10.1038/s41467-018-04558-1. There are parallels between the studies and the conclusions even though that study focussed on eQTL across brain and increased power from blood, and here focus is immune cell types including blood.

i) That study approached the power issue through simulation and provided a statistic (MeCS) software to estimate correlation of effect size accounting for s.e. Maybe mash does something similar

Thank you for pointing this out, we were unaware of the Qi et al paper and we have now added a reference to the work in our manuscript page 3. *“These data allowed us to evaluate the cell-type-specificity of eQTLs while considering limited statistical power due to small sample size (Urbut et al. 2019, Qi et al, 2018)”.*

In the MeCS paper, the statistic r_b_ describes the correlation of effect sizes between two tissues while considering the error in original effect sizes. Similarly, mash re-estimates effect sizes by leveraging the correlation structures among tissues and reports posterior effects or Z-scores. Mash also reports local false sign rates to allow assessment of significance for each QTL in each tissue. The posteriors from mash are “shrunk” estimates, which also have smaller standard error., but it is not clear whether it offers a better estimation of correlation in effect sizes. Answering this question may require some detailed benchmarking of the mash method, which is outside the scope of our manuscript. However, compared to mash, the method presented in Qi et al. does not provide re-estimates of effect sizes nor the level of significance.

As expected, mash also increases effect size correlation (calculated as Pearson’s) between pairs of cell-types, consistent with increased sharing of QTL using mash.

ii) Their Figure 3, x axis difference in eQTL effect between two tissues vs y-axis difference in mean gene expression for the gene between two tissues was very interesting to me. It would be interesting to see if this relationship replicates. At first sight (Figure 2d) it seems not, but this is a more condensed analysis.

In Figure 2D, we asked whether the expression levels of eGenes were significantly higher in the cell-type in which the eGenes is specifically detected. We do not directly test the correlation between differences in eQTL effect sizes and differences in gene expression level. While these two questions are related, we note that Qi et al. re-scaled their cis-eQTLs “based on the expression level per gene in SD units so that the correlation was not confounded by the mean–variance relationship in gene expression.”

We did not account for the mean-variance relationship, as the chief goal of our analysis was to determine factors that contribute to the cell-type-specificity of eQTLs -- regardless of whether driven by biology or by differences in statistical power. We find in our analysis shown in Figure

2D that differences in gene expression levels can indeed partly explain cell-type-specificity, likely because the eGenes are not expressed highly enough to be detected in other cell-types.

We decided not to add this technical explanation to the revised version of the manuscript as it would disrupt the flow of the manuscript, which we have worked hard to improve.

iii) In comparing eQTL effect sizes between two tissues, they first identified eQTL in a third tissue and used those SNPs as the basis for comparing eQTLs in the two tissues, arguing this could avoid winner's curse. I am not suggesting a massive re-analysis as I think you have already done so much. Rather, I just wondered if this paper could offer anything to further improve interpretation. If not, then no need to consider further.

Thank you for this comment. To clarify, we used DGN, a third dataset, to ascertain the eQTL effect sizes of (i) eQTL that were only found in eQTLGEN, (ii) eQTLs specific to BLUEPRINT, and (iii) eQTLs shared between eQTLGEN and BLUEPRINT. The Winner’s Curse effect in the context of QTL mapping is the phenomenon where the effect size of the lead SNP (with smallest p-value) found using linear regressions that test all SNPs, one by one, will result in overestimating the true effect size of the causal SNP. To avoid the winner’s curse effect, one can take an independent dataset to re-estimate the effect of the lead SNP. In this case, we re-estimated the effect sizes in DGN and showed that the effect sizes of eQTLGen-specific eQTLs are much smaller than those specific to BLUEPRINT, or shared. Because DGN is meta-analyzed as part of the eQTLGen data, DGN may not fully remove Winner’s Curse effect. However, given that the DGN datasets represent only 922 samples out of > 30,000 samples meta-analyzed, we believe that the effect is minimal.

We have modified our manuscript from *“To avoid the Winner’s curse effect, the effect sizes were ascertained using the DGN dataset.”* to “ *To reduce the Winner’s curse effect, the effect sizes were ascertained using the DGN dataset* .”

3) are all the eQTL meta-analysed results available for download (including with eQTLgen), All of the significant eQTL and sQTL are available to download as Supplementary Data. eQTLGen summary statistics is available to download from the eQTL website, which has been cited in our manuscript. We have now added a supplementary table (Supplementary Table 8) describing all the data that were analyzed and generated in our study and where to find them. We also included the URL where data is deposited in the “Availability of data and materials” section as required by the journal.

Minor points

1) "Interestingly, we observed that the rates of colocalization between GWAS loci and both an eQTL and an sQTL can vary substantially across traits, ranging from 4.5% for systemic lupus erythematosus (SLE) to 28.6% for basophil percentages of granulocyte (BASO%GRAN) (average: 17.2%)." This statement is hard to benchmark because of the impact of GWAS

sample size. As far as I can see you emphasise consequence of eQTL sample size but not GWAS sample size, but maybe I missed.

We agree that both eQTL sample size and GWAS sample size can impact the proportion of colocalized GWAS loci. However, since we are using the same eQTL study in our colocalization analysis, this difference in percentages cannot be due to eQTL sample size. As you noted, the GWAS sample sizes are different across traits and may impact overall rates of colocalization. However, we have not seen any correlation between sample size and fraction of the colocalized loci. We have added Supplementary Figure 7 to show colocalization % with eQTLs and sQTLs as a function of GWAS sample sizes and of total significant loci.

We now mention this in the revised text and clarify our assertion:

*“Interestingly, the rates of colocalization were not correlated with GWAS sample size nor the number of significant loci, and thus the variation in colocalization rates cannot be attributed to differences in GWAS power (Supplementary Figure 7) .”*

2) Page 5:" DICE also performed in vitro activation of CD4+ and CD8+ T cells using CD3/CD28 antibodies." On first reading a reader isn't familiar with acronyms so this would be better phrased as "The DICE data set also included gene expression data after in vitro activation..."

We have edited the sentence to *“The DICE dataset also includes RNA-seq data from CD4+ and*

*CD8+ T cells that have been activated in vitro by engaging T-cell receptor (TCR) complex using*

*CD3/CD28 antibodies”*

3) Page 8: "This observation suggests that activated CD4+ and CD8+ T cells share similar gene expression programs upon activation, and that differences in genetic effects on gene regulation exist between activated and non-activated cells." This is interesting. Are there gene lists that could be provided. Eg the summary data behind Figure 2, with a statistic for ranking differences?

We have added the genes behind Figure 2c to the Supplementary Table 3.

Page 14: Readers will want to be able to identify these "We observed that the top eQTLs are often in high LD with the lead GWAS SNP for multiple cell-groups (rows) when the eQTLs were determined to have shared effects. By contrast, for the eQTLs we inferred to have a cell-type-specific effect, the patterns are strikingly different as the most significant eQTLs are more likely to be in lower r2-bins in most cell-types' Are these results available in the supplementary files.

Thank you for your interest. We have added a Supplementary Figure (14) similar to Figure 4 d-e including all 15 cell-types from DICE, and not just the collapsed cell categories to show the more detailed picture of P-value distribution across r2-bins. We have also added a

Supplementary Table (5-6) for the raw P-values that were used in plotting the figures along with gene names.

4)Page 11. "Ten and fifteen loci colocalized with only eQTLs or sQTLs, respectively, while an additional 25 loci colocalized with both eQTLs and sQTLs. In total, 46% of loci colocalized with an eQTL, an sQTL, or both" So are the eQTL and sQTL SNPs in LD or independent?

For the 25 GWAS loci that colocalized with both an eQTL and a sQTL, we calculated the LD (r^2^ ) between colocalized eQTL SNP and sQTL SNP for the same loci. Several GWAS loci colocalized with more than one eQTL or sQTL, we therefore tested 118 pairs of r^2^ for the 25 loci. Here, we defined colocalize QTL SNP as the SNP with the largest per-SNP PP4 value in the locus. Although the exact eQTL and sQTL SNPs may differ, they are almost expected to be tagging the same causal variant. As expected, the SNPs are in close LD (median: 0.95; mean: 0.99; with only one exception: 0.06, the remaining are all above 0.6). Indeed, since colocalized QTLs are usually in high LD with the GWAS lead SNP, it is not surprising that the eQTL and sQTL that colocalize with the same GWAS lead SNP are also in high LD.

We have decided not to add this explanation to ensure that our manuscript is as streamlined as possible (see comments from reviewer 2).

5) Page 18. Note if DGN is part of eQTLgen

We thank the reviewer for pointing this out. DGN is one of the consortium members of eQTLGen. We have now noted this in the revised manuscript. *“Of note, the DGN dataset is also included in eQTLGen meta-analysis, but does not alter the interpretation of any of our analyses.” and “As an additional test, we asked how many uncolocalized GWAS loci could be colocalized using eQTL summary statistics from eQTLGen, which were obtained from a meta-analysis of 31,684 whole blood samples Vosa et al 2018, including the 922 DGN samples”.*

6) Fig 2b, where "Sharing of eQTLs (left) and sQTLs (right) by magnitude. " isnt very unclear what is plotted. This is where the MeCSstatistic may be useful. Although I have now found in the MashR methods section.

We have now clarified this in the revised version of the manuscript: *“Sharing of eQTLs (left) and sQTLs (right) by magnitude (Fold difference in QTL effect size less than 2)”*

7) "To avoid reads mapping with allelic bias, we used WASP [58] as implemented in STAR 2.6.0 by providing the corresponding genotype data. This is an important step as we found a substantial increase in the number of false positive splicing QTL due to allelic bias in read mapping." Please add another sentence to clarify further.

We have now clarified this in the revised version of the manuscript: *“ This is an important step as we found a substantial increase in the number of false positive splicing QTL due to allelic bias in read mapping. Indeed, when reads representing different alleles map to different regions of the genome, QTL mapping will be susceptible to identifying spurious associations between the alleles and read coverage at those genomic regions.”*

Reviewer #2:

The authors perform systematic colocalisation analysis between several eQTL and sQTL datasets and 72 GWAS traits, with a particular focus on the autoimmune diseases. They find that many more GWAS signals colocalised with eQTLs than in previous research, and especially with sQTLs and that, moreover, that cell-specific eQTLs accounted to much fewer colocalisations than previously reported. Reasons for these discrepancies are postulated and studied. Additionally, authors investigate potential mechanisms through which the 60% of non-colocalised GWAS signals may affect disease susceptibility and mechanisms. As we are moving away from the GWAS era, the manuscript addresses interesting questions about disease causing mechanisms and systemic properties of gene expressions in multiple cell types. Interesting results are presented. However, I have some reservations about the manuscript in its current format.

A major concern is the structure and length of the manuscript---it appears to be overly long and is hard to follow. An awful lot of work has been done, but a lack of coherent presentation obscures the main messages.

Thank you for your comments. We worked hard to streamline the parts you highlighted in order to improve the clarity of our manuscript. We have also moved several sections of the main text to the supplementary notes again to improve flow and clarity.

They appear to be

- greater sharing of QTLs across cell types. This is based on inference by mash, on which I have some major methodological concerns (see below).

See below for our reply.

- Stronger colocalisation of QTL and GWAS effects, particularly for autoimmune traits, driven by inclusion of sQTL data. The presentation here is very unclear. When QTLs colocalise with a % of GWAS traits, what is the denominator? The phrase "we obtained association between all colocalized eQTL SNPs" on page 11... colocalised with what?

We only reported colocalization rates as a percentage of GWAS loci for one trait, but not as a percentage of GWAS traits. Therefore, the denominator is the number of significant GWAS loci for each trait. We described the procedure of identifying these GWAS loci in Methods section. In the sentence “we obtained association between all colocalized eQTL SNPs”, we extracted from GTEx data the effect sizes and p-values of all colocalized eQTL in DICE.

We have now re-written the sentences surrounding these to improve clarity: *“ We computed the colocalization rate for each GWAS as the percentage of GWAS loci that show evidence of colocalization out of the total number of associated loci in the GWAS (Methods)”*

We have also clarified that the colocalized eQTL SNPs were with non-immune GWAS loci: *“To identify trait-associated variants that likely act through immune cell-types, we obtained eQTL p-values from relevant GTEx tissues for the DICE eQTL SNPs that colocalized with non-immune GWAS loci. Using these GTEx eQTL p-values, asked about the proportion of immune regulatory eQTLs that colocalize with non-immune GWAS loci that do not show any effects on gene expression levels in GTEx tissues that are most relevant to each trait.”* Importantly, we moved this entire analysis of non-immune traits to the Supplementary Notes to improve coherence and reduce the length of our manuscript.

The inference that colocalisation of eQTL signals in whole blood and hypertension is used to support immune involvement in hypertension - but could it not also reflect sharing of QTLs between cell types as the authors propose?

As you point out, colocalization of eQTLs in immune cells with hypertension GWAS could be due to sharing of eQTLs with true causal tissue. However, we attempted to address this concern by using GTEx data. Specifically, because the GTEx consortium contains a wide range of tissues, we reasoned that if a colocalized eQTL in immune cells is not a significant eQTL in any of the GTEx tissues, then this suggests that the eQTL might be causal in immune cells. Again, we moved this entire analysis to the Supplementary Notes to improve coherence and reduce the length of our manuscript.

- Limited cell state specific regulatory effects: is this not a restatement of the first major point?

In the section of “Limited cell state specific regulatory effects”, we focused on stimulated immune cells, which is what we mean by “cell states”, in contrast to “cell types”. The reason for this distinction is because multiple previous papers specifically proposed that studying stimulated immune cells (rather than additional cell-types) provides novel insight to gene regulatory programmes relevant to autoimmune diseases (Calderon et al. and Soskic et al.).

In our work, we found that unstimulated immune cells share a large proportion of QTLs with stimulated immune cells. Indeed, when we tested for the enrichment of GWAS loci that do not overlap with e/sQTLs in unstimulated immune cells, we found that e/sQTLs in stimulated cells showed either no or weak enrichment among GWAS loci. This observation suggests that stimulated immune cells show limited cell state specific regulatory effects that are relevant to the

interpretation of immune-related GWAS variants. Although the interpretation is largely similar to our first major point as you have noted, we have decided to emphasize it to a larger extent given the previous studies proposing the importance of studying the genetic effects on gene expression in stimulated immune cells.

Methodological concern on mash Reading

[https://urldefense.proofpoint.com/v2/url?u=https-3A stephenslab.github.io_mashr_articles_intr](https://urldefense.proofpoint.com/v2/url?u=https-3A__stephenslab.github.io_mashr_articles_intro-5Fmash.html&d=DwIGaQ&c=vh6FgFnduejNhPPD0fl_yRaSfZy8CWbWnIf4XJhSqx8&r=Y4r1_XDXu4yYdegfBIAiGEOf3a2Qz0JY3Z5HybxTDto&m=LZRrrdfSL_l_8XAfXmLfLiDegEdNws6HJA1oOJOQwUg&s=Muc6qeq_FYZISe_BkcG7UtMo4ifmX4CtdL3_1pit928&e=)  [o-5Fmash.html&d=DwIGaQ&c=vh6FgFnduejNhPPD0fl_yRaSfZy8CWbWnIf4XJhSqx8&r=Y4r1_ XDXu4yYdegfBIAiGEOf3a2Qz0JY3Z5HybxTDto&m=LZRrrdfSL_l_8XAfXmLfLiDegEdNws6HJA](https://urldefense.proofpoint.com/v2/url?u=https-3A__stephenslab.github.io_mashr_articles_intro-5Fmash.html&d=DwIGaQ&c=vh6FgFnduejNhPPD0fl_yRaSfZy8CWbWnIf4XJhSqx8&r=Y4r1_XDXu4yYdegfBIAiGEOf3a2Qz0JY3Z5HybxTDto&m=LZRrrdfSL_l_8XAfXmLfLiDegEdNws6HJA1oOJOQwUg&s=Muc6qeq_FYZISe_BkcG7UtMo4ifmX4CtdL3_1pit928&e=)  [1oOJOQwUg&s=Muc6qeq_FYZISe_BkcG7UtMo4ifmX4CtdL3_1pit928&e=](https://urldefense.proofpoint.com/v2/url?u=https-3A__stephenslab.github.io_mashr_articles_intro-5Fmash.html&d=DwIGaQ&c=vh6FgFnduejNhPPD0fl_yRaSfZy8CWbWnIf4XJhSqx8&r=Y4r1_XDXu4yYdegfBIAiGEOf3a2Qz0JY3Z5HybxTDto&m=LZRrrdfSL_l_8XAfXmLfLiDegEdNws6HJA1oOJOQwUg&s=Muc6qeq_FYZISe_BkcG7UtMo4ifmX4CtdL3_1pit928&e=) the mash instructions say "The Crucial Rule: Step 3 (fitting the mash model) must be performed with either all the tests you performed, or - if that causes computational challenges - a large random subset of tests. In particular, you must not use only a set of selected "significant" or "strong" tests in step 3. " Yet in the methods here, the authors say they ran mash using SNPs with the smallest p values across all tested SNPs and cell types for each feature. Does this claim of sharing persist if mash is run correctly? How does this impact all subsequent results?

We thank the reviewer for raising this important question. Indeed, mash uses the full dataset or a large random subset to fit the model. Specifically, it is recommended to (1) learn the correlation structure among null tests using a random subset of all tests, (2) learn data-driven covariance matrices using strong tests, which we describe in our manuscript as the tests with “smallest p values across all tested SNPs and cell types for each feature”, (3) fit the mashr model to the random tests, and (4) compute posterior summaries on the strong tests, which gives LFSR and posterior effect sizes that we discussed in the manuscript (<https://stephenslab.github.io/mashr/articles/eQTL_outline.html)>. This is the analysis pipeline that we used in our study and we therefore believe that our results from the mash analysis are correct.

That being said, we agree that our description in the Methods was confusing and inaccurate, and we have updated the relevant section in the revised manuscript:

*“To quantify the sharing of eQTLs and sQTLs in the DICE dataset, we followed the workflow provided by the authors of MashR (*[*https://github.com/stephenslab/gtexresults)*](https://github.com/stephenslab/gtexresults)) *that was previously described in Urbut et al. 2018. Briefly, standard errors of QTL effect sizes were calculated from FastQTL nominal output, which were used together with effect sizes as the input for mash. To quantify the correlation structure of the null tests, 30% of all tests were randomly sampled (referred to as the “random” set). To obtain a confident set of QTLs for each feature (gene or intron), the SNP with the smallest P-value across all tested SNPs and all cell-types were extracted for each feature. This resulted in a feature-by-sample matrix of effect sizes and their standard errors without missing values referred to as the “strong” set. For eQTLs, we included all protein coding genes. For sQTLs, we included all introns. Data-driven covariance matrices were calculated from the “strong” set. We then built a mash model using the “random” set with the exchange effects (EE) mode to estimate the priors. This model was then applied to the “strong” set to calculate the posterior mean effect sizes mash effect sizes. Significant QTLs after mash analysis were feature-SNP pairs with local false sign rate (LFSR) below 0.05, as suggested by Urbut et al. 2018.”*

Even if mash is run correctly, given that mash assumes effect estimates are correlated, does it have a tendency to over-call sharing of effects? This is important as the accuracy of mash was primarily assessed on its ability to *detect* true associations. Evidence of over-calling of sharing is apparent in Table 1 of Urbut et al. If there is over-calling, how does this impact conclusions?

In Table 1 of Urbut et al., the “shared by sign” values might seem overly high, but the “shared by magnitude” values are much lower, especially for all tissues and non-brain tissues. Therefore, we believe that the problem of over-calling does not significantly affect our results in Figure 2b (left). Furthermore, users may choose a more stringent cutoff value for difference in magnitude (here we used 2, the same as Urbut et al.), which would lead to more conservative estimation in QTL sharing. The implication of this is simply that GWAS SNPs can have differences in their effect sizes on gene expression levels depending on the cell-type, but the eQTL effects should be detected in all cell-types given large enough sample sizes. We therefore believe that this will not affect our overall conclusion that cell-type-specificity of eQTL effects is not a major reason for the lack of colocalization between autoimmune GWAS loci and eQTLs.

Minor issues include typos and presentational issues, e.g.:

the mash method is mentioned without description in Figure 1 and is
not introduced until page 6 despite its application to the problem
being part of the novel contribution of the paper.

We have added a sentence in the figure legend to describe the mash method: *“Sharing of QTLs among cell-types were quantified using mash (Urbut et al., 2018) a statistical method that leverages the correlation structure of QTL effect sizes across multiple samples to re-estimate QTL effect in each sample.”*

p 1 sQTL abbreviation not formally defined (should be on line 17/18) p1 line 17/18. QTLs should be eQTLs.

Thank you for noting this mistake, we have now fixed this in the revised text.

p 2 line 2. Citation needed

We have added a citation (Hindorff et al., 2009) in the revised text.

p 3 line 26. should be "40.4% *of* GWAS loc"

Thank you again for noting this, we have now fixed this.

Caption to Figure 1a. "four large immune RNA-seq datasets"---these were specifically previously described as datasets of different sizes with only two of them as large (p 3 lines 18-22).

Thank you for this comment. We initially characterized the DICE dataset as large in the sense that it contains many cell types. We have now removed “large” from the sentence in the caption.

Figure 1b. Would be nice to label the LHS part of the graph with DICE to make labelling self-sufficient, Grammatically incorrect main title of the graph.

Thank you for this suggestion, we have now updated Figure 1.

p 5 line 3. eQTL abbreviation already explained on p 2 line 8, whilst sQTL has already been used without being defined.

We have addressed this in the text.

page 6. p 6 line 18. LFSR not defined.

We have now defined this in the updated text.

p 20 line 2. I refer the authors to a recent paper

[https://urldefense.proofpoint.com/v2/url?u=https-3A journals.plos.org_plosgenetics_article-3Fi](https://urldefense.proofpoint.com/v2/url?u=https-3A__journals.plos.org_plosgenetics_article-3Fid-3D10.1371_journal.pgen.1008720-26rev-3D2&d=DwIGaQ&c=vh6FgFnduejNhPPD0fl_yRaSfZy8CWbWnIf4XJhSqx8&r=Y4r1_XDXu4yYdegfBIAiGEOf3a2Qz0JY3Z5HybxTDto&m=LZRrrdfSL_l_8XAfXmLfLiDegEdNws6HJA1oOJOQwUg&s=dnY_shGxU5PQsqk-yoaSFx7MbuA2uk2Az-UXZ0j6lrI&e=)  [d-3D10.1371_journal.pgen.1008720-26rev-3D2&d=DwIGaQ&c=vh6FgFnduejNhPPD0fl_yRaSfZ y8CWbWnIf4XJhSqx8&r=Y4r1_XDXu4yYdegfBIAiGEOf3a2Qz0JY3Z5HybxTDto&m=LZRrrdfSL](https://urldefense.proofpoint.com/v2/url?u=https-3A__journals.plos.org_plosgenetics_article-3Fid-3D10.1371_journal.pgen.1008720-26rev-3D2&d=DwIGaQ&c=vh6FgFnduejNhPPD0fl_yRaSfZy8CWbWnIf4XJhSqx8&r=Y4r1_XDXu4yYdegfBIAiGEOf3a2Qz0JY3Z5HybxTDto&m=LZRrrdfSL_l_8XAfXmLfLiDegEdNws6HJA1oOJOQwUg&s=dnY_shGxU5PQsqk-yoaSFx7MbuA2uk2Az-UXZ0j6lrI&e=)  [_l_8XAfXmLfLiDegEdNws6HJA1oOJOQwUg&s=dnY_shGxU5PQsqk-yoaSFx7MbuA2uk2Az-U](https://urldefense.proofpoint.com/v2/url?u=https-3A__journals.plos.org_plosgenetics_article-3Fid-3D10.1371_journal.pgen.1008720-26rev-3D2&d=DwIGaQ&c=vh6FgFnduejNhPPD0fl_yRaSfZy8CWbWnIf4XJhSqx8&r=Y4r1_XDXu4yYdegfBIAiGEOf3a2Qz0JY3Z5HybxTDto&m=LZRrrdfSL_l_8XAfXmLfLiDegEdNws6HJA1oOJOQwUg&s=dnY_shGxU5PQsqk-yoaSFx7MbuA2uk2Az-UXZ0j6lrI&e=)  [XZ0j6lrI&e=](https://urldefense.proofpoint.com/v2/url?u=https-3A__journals.plos.org_plosgenetics_article-3Fid-3D10.1371_journal.pgen.1008720-26rev-3D2&d=DwIGaQ&c=vh6FgFnduejNhPPD0fl_yRaSfZy8CWbWnIf4XJhSqx8&r=Y4r1_XDXu4yYdegfBIAiGEOf3a2Qz0JY3Z5HybxTDto&m=LZRrrdfSL_l_8XAfXmLfLiDegEdNws6HJA1oOJOQwUg&s=dnY_shGxU5PQsqk-yoaSFx7MbuA2uk2Az-UXZ0j6lrI&e=)

for analysis of sensitivity to prior parameters of the coloc method.

Thank you for this. One major conclusion from Wallace’s work is that misspecification of prior parameters can substantially alter posterior inference in coloc. In our work, we spend some time playing with different colocalization approaches including approaches that attempt to come up with better prior parameters (HyPrColoc, as presented in our Supplementary Notes, and DAPR, which we did not show). However, our general conclusion is that the different methods performed at a similar level (more colocalizations for some traits, and fewer in some other traits). Given the popularity of COLOC, we decided to stick with COLOC with uniform prior, but we acknowledge that statistical improvement of the colocalization method may reveal some new colocalizations.

We have now added mention of the Wallace study in our supplementary notes section (“Robustness of colocalization”): *“A recent study on COLOC (Wallace, 2020) reported that misspecification of prior parameters can heavily impact the inferred posterior probability of colocalization.”*

**Second round of review**

**Reviewer 1**

The authors have addressed my comments
